# Clinical observational study on the efficacy of induction chemotherapy sequential concurrent radiotherapy combined with targeted therapy in patients with locally advanced EGFR-positive nasopharyngeal carcinoma: prediction model construction and efficacy testing

**DOI:** 10.1007/s00405-023-08157-9

**Published:** 2023-08-02

**Authors:** Yuanyuan Luo, XueJing Xiang, XiaoJie Ma

**Affiliations:** 1 Affiliated Hospital of North Sichuan Medical College, Nanchong, 637000 China; 2 North Sichuan Medical College, Nanchong, 637000 China

**Keywords:** Nasopharyngeal tumor, Prognosis, Induction chemotherapy sequential concurrent radiotherapy, Targeted therapy

## Abstract

**Objective:**

To establish a nomogram for prediction of prognosis in EGFR-positive advanced nasopharyngeal carcinoma (NPC) patients who were treated with induction chemotherapy (IC) and concurrent chemoradiotherapy (CCRT). The clinical data of 124 NPC patients who received IC sequential CCRT combined with targeted therapy at the Department of Oncology of the Affiliated Hospital of North Sichuan Medical College between June 2017 and September 2022 were retrospectively reviewed. Logistic regression analysis was used to identify the prognostic factors for building the nomogram.

**Results:**

Multifactorial regression analysis showed that the use of targeted drugs and T stage were independent factors of prognosis (*p* < 0.05) and the equation *Y* = 0.476 + 2.733X1 + − 0.758 × 2 (*Y* = efficacy, X1 = targeted drug therapy, X2 = T stage) was obtained. Then, a prognostic nomogram prediction model was constructed. The prediction model was validated internally for 1000 times using the Bootstrap resampling method with an accuracy of 79.29%. The calibration curve suggests that the predicted values fit well with the true values. The clinical decision curve (DCA) shows that the model has good clinical predictive value.

**Conclusion:**

The use of targeted therapy significantly improved the prognosis of patients with EGFR-positive advanced NPC. For advanced NPC patients with T1 and T2 stages, IC sequenced with CCRT is more effective, and the addition of targeted therapy can further improve patients’ prognosis. For advanced NPC patients with T3 and T4 stages, IC sequenced with CCRT is ineffective, and the addition of targeted therapy can significantly improve patient prognosis.

## Introduction

Nasopharyngeal carcinoma (NPC) is a malignant tumor that originates from the nasopharyngeal epithelium and is highly prevalent in Southeast Asia, particularly in southern China [1–3]. The latest guideline recommended that concurrent chemoradiotherapy (CCRT) with induction chemotherapy (IC) is recommended as the first-line treatment for locoregionally advanced NPC [4–6]. Although NPC is sensitive to radiotherapy and chemotherapy, the treatment failure rate remains high due to the development of local recurrence, distant metastasis [7, 8]. However, patients with local recurrence and distant metastases have a 5-year survival rate of only 10% with current therapies [9, 10]. Given the poor prognosis, effective and accessible prognostic models for NPC patients are urgently needed to assess patient prognosis for treatment optimization.

EGFR is a member of a family of transmembrane protein kinase receptors known as erbB or HER receptors: EGFR (HER1 or erbB1), erbB2 (HER2), erbB3 (HER3), and erb4 (HER4), which is vital to epithelial cell biology [11]. HER2 is overexpressed in a wide variety of malignancies, including nasopharyngeal carcinoma, breast cancer, bladder cancer, and lung cancer. Several studies have shown that more than 80% of patients with nasopharyngeal cancer (NPC) have epidermal growth factor receptor (EGFR) overexpression [12, 13]. The amplification, mutation, and autocrine, paracrine, juxtacrine and/or endocrine activation are responsible for the increased activity of EGFR in NPC. For patients with advanced NPC who are EGFR mutation positive, combination therapy of IC, CRRT, and targeted therapy is recommended.

A humanized anti-EGFR monoclonal antibody called nimotuzumab binds to the EGFR extracellular domain and prevents EGF binding and signaling, aiming to reduce immunoreactivity and enhance radiosensitivity [14]. For the prognosis of NPC patients, there were numerous prognostic models available. However, as far as we can tell, there is currently a lack of a model that can accurately predict the prognosis of patients with EGFR-positive NPC, which is coupled with targeted drugs. Therefore, we aim to establish a prognostic model to predict the prognosis for EGFR-positive NPC patients with IC sequential CCRT combined with targeted therapy and optimize the clinical treatment plan.

## Information and methods

### General information

We retrospectively analyzed 124 patients with EGFR-positive NPC between June 2017 and September 2022 at the Department of Oncology of the Affiliated Hospital of North Sichuan Medical College. The inclusion criteria were as follows: (1) patients were pathologically diagnosed with NPC and had positive immunohistochemical results for EGFR; (2) these patients were clinically staged according to the 8th edition of the AJCC/UICC TNM staging system; (3) patients had not received prior radiotherapy; (4) these patients were treated with targeted therapies; (5) they did not have active acute or chronic infectious diseases and autoimmune diseases; and (6) these patients had complete clinical data. We collected the following data: age, gender, tumor staging, pathology type, smoking and alcohol history, EBV counts, height, weight, blood counts, neutrophil to lymphocyte ratio (NLR), platelet to lymphocyte ratio (PLR), lymphocyte to monocyte ratio (LMR), and treatment regimen. This study was approved by the Hospital Ethics Committee in the Hospital of Sichuan North Medical College in China.

### Treatment methods

Induction chemotherapy (IC): paclitaxel 60 mg/m^2^ intravenously once a day, 5-fluorouracil 500 mg/m^2^ intravenously once a day on days 1–3, and cisplatin 30 mg/m^2^ once a day on each cycle.

Concurrent radiotherapy (CCRT): patients were positioned in the supine position with head, neck, and shoulder thermoplastic molds, and the tumor was localized using CT-enhanced scans from the top of the head to the bifurcation of the tracheal ramus. The radiation dose was 60–68 Gy for the cervical metastatic lymph nodes, 70–72 Gy for the major foci in the nasopharynx, 60–64 Gy for the high-risk region, and 54–60 Gy for the preventive region. It took 6–7 weeks to complete 31–33 sessions at a rate of one treatment per day, five days a week. During irradiation, nedaplatin 30 mg/m^2^ was administered once per week.

Targeted therapy: for EGFR-positive patients, nitrozumab injection 200 mg was given intravenously once a week during concurrent radiotherapy with the consent of the patient.

### Evaluation metrics

Recent outcomes were assessed according to the solid tumor efficacy evaluation index [15]: complete remission (CR), partial remission (PR), stable disease (SD), and disease progression (PD). CR group = CR, non-CR group = PR + SD.

The horizontal axis of the clinical decision curve (DCA) is the threshold probability and the vertical axis is the net benefit (benefit for true positives—loss for false positives), the threshold probability is the probability that the patient will choose to receive the treatment and the DCA curve is the change in net benefit for the model intervention scenario when the threshold probability changes [11].

### Statistical processing

Data were analyzed using SPSS25.0 and Graphpad prism 9 software. nomogram models were constructed and tested using R 4.2.1 software and the RMS program package. The measurement data were first tested for normality (*x* ± *s*). The *t* test was used for the measurement data; the *χ*^2^ test or Fisher’s exact test was used for the count data. The logistic regression model was used to construct prediction models for the indicators that were significant for *d* single factor, and C-index, AUC, calibration curve, and DCA curve were used to further validate the models. *p* < 0.05 is statistically significant.

## Results

### General information of patients

Among the 124 patients in this study, there were 72 patients with CR, 43 patients with PR and 9 patients with SD; 72 patients in the CR group and 52 patients in the non-CR group. The mean age of the patients was (52.52 ± 11.89) years, the mean age of patients in CR group was (51.12 ± 11.63) years, and the mean age of patients in non-CR group was (54.44 ± 11.92) years, and the specific clinical characteristics are shown in Table 1.

**Table 1:** General clinical features of patients with EGFR-positive nasopharyngeal carcinoma

Clinical characteristics	*N*%	*p*
Sex		0.8471
Male	83 (66.9)	
Female	41 (33.1)	
Age		0.3448
< 50	45 (36.3)	
≥ 50	79 (63.7)	
Staging		0.8448
III	61 (49.2)	
IVa	63 (50.8)	
T stage		< 0.01
T1–2	57 (46.0)	
T3–4	67 (54.0)	
N stage		0.1795
N0–2	90 (72.6)	
N3	34 (27.4)	
Pathological type		> 0.999
Keratinized type	6 (4.8)	
Non-keratinized	118 (95.2)	
Smoking		0.0887
Yes	49 (39.5)	
No	75 (60.5)	
Drinking		0.6832
Yes	33 (26.6)	
No	91 (73.4)	

### Univariate analysis of patient clinical characteristics and outcome

Univariate analysis showed that T stage, targeted drug therapy, induction chemotherapy cycles, neutrophils, monocytes, platelets, hemoglobin, NLR, PLR, and LMR were associated with the recent outcomes of EGFR-positive NPC patients (*p* < 0.05), as shown in Table 2.

**Table 2:** Results of univariate analysis

Results of one-way analysis	CR group (*n* = 72)	No-CR group (*n* = 52)	*p*
Age			0.3448
< 50	29 (40.3)	16 (30.8)	
≥ 50	43 (59.7)	36 (69.2)	
Sex			0.8471
Male	49 (68.1)	34 (65.4)	
Female	23 (31.9)	18 (34.6)	
Staging			0.8448
III	38 (52.8)	23 (44.2)	
IVa	34 (47.2)	29 (55.8)	
T stage			< 0.01
T1–2	44 (61.1)	13 (25)	
T3–4	28 (38.9)	39 (75)	
N stage			0.1795
N0–2	53 (73.6)	37 (71.2)	
N3	19 (26.4)	15 (28.8)	
Pathological type			
Keratinized type	5 (6.9)	1 (1.9)	> 0.999
Non-keratinized	67 (93.1)	51 (98.1)	
Body mass index (BMI)			0.3914
< 24.9	51 (70.8)	38 (73.1)	
≥ 24.9	21 (29.2)	14 (26.9)	
IC cycles			0.03
1 cycle	11 (15.3)	3 (5.8)	
2–3 cycles	61 (84.7)	49 (94.2)	
IC regimen			0.18
Doublet	46 (63.9)	29 (55.8)	
Triplet	26 (36.1)	23 (44.2)	
Targeted therapy			
Yes	61 (83.6)	19 (36.5)	< 0.01
No	11 (16.4)	33 (63.5)	
Smoking			0.0887
Yes	25 (34.7)	24 (46.2)	
No	47 (65.3)	28 (53.8)	
Drinking			0.6832
Yes	18 (25)	15 (28.8)	
None	54 (75)	37 (71.2)	
EBV counts (copies/L)			
< 400	64 (88.9)	41 (78.8)	0.1380
≥ 400	8 (11.1)	11 (21.2)	
Leukocyte (10^9/L)			0.3819
Normal (4–10)	43 (58.9)	45 (67.2)	
Abnormal (< 4or > 10)	30 (41.1)	22 (32.8)	
Medium granulocytes (10^9/L)			< 0.001
Normal (1.5–8)	53 (72.6)	54 (80.6)	
Abnormal (< 1.5 or > 8)	20 (27.4)	13 (19.4)	
Lymphocytes (10^9/L)			0.1793
Normal (0.8–4)	44 (60.3)	43 (64.2)	
Abnormal (< 0.8 or > 4)	29 (39.7)	24 (35.8)	
Monocytes (10^9/L)			< 0.001
Normal (0.12–0.8)	58 (79.5)	52 (77.6)	
Abnormal (< 0.12 or > 0.8)	15 (20.5)	15 (22.4)	
Platelets (10^9/L)			< 0.001
Normal (100–300)	64 (87.7)	60 (89.6)	
Abnormal (< 100 or > 300)	9 (12.3)	7 (10.4)	
Hemoglobin (g/L)			< 0.001
Normal (Man 120–160, Woman 110–150)	47 (35.6)	43 (64.2)	
Abnormal (Man < 120 or > 160, Woman < 110 or > 150)	26 (64.4)	24 (35.8)	
NLR			< 0.001
≥ 9.8198	14 (19.4)	6 (11.5)	< 0.001
< 9.8198	58 (80.2)	46 (88.5)	
LMR			< 0.001
≥ 0.2514	42 (58.3)	25 (48.1)	
< 0.2514	30 (41.7)	27 (51.9)	
PLR			< 0.001
≥ 389.122	10 (13.9)	5 (9.6)	
< 389.122	62 (86.1)	47 (90.4)	

*NLR* neutrophils/lymphocytes, *PLR* platelets/lymphocytes, *LMR* lymphocytes/monocytes

### Construction of a clinical prediction model for the efficacy of IC sequential CCRT combined with targeted therapy for EGFR-positive NPC

**Fig. 1:**
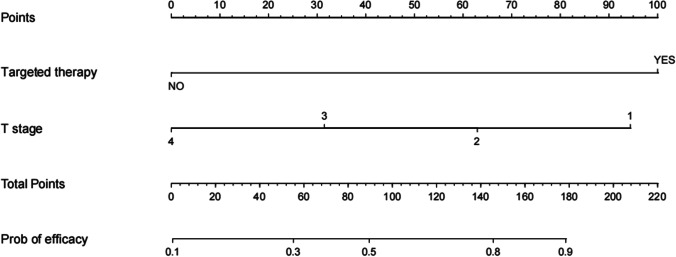
A nomogram model for predicting the efficacy of EGFR-positive NPC IC sequential CCRT combined with targeted therapy

Since NLR and PLR are derived from neutrophils and platelets by simple arithmetic, there is a linear correlation. Therefore, neutrophils and platelets were not included in the multifactorial regression, which finally showed that the targeted therapy and T stage were independent factors of prognosis (*p* < 0.05) (Table 3).The prediction model equation for the efficacy of IC sequential CCRT combined with targeted therapy in EGFR-positive NPC patients was obtained as *Y* = 0.3199 + 2.494X1 + − 0.7547 × 2 (*Y* = efficacy, X1 = targeted therapy, X2 = T stage). It is also presented as nomogram (Fig. 1).

**Table 3:** Logistics analysis screening independent factors

Clinical data	*β*	SE	*p*	OR (95%CI)
Targeted therapy	2.494	0.5004	< 0.0001	1.562 to 3.540
IC cycles	− 0.1796	0.3749	0.6319	− 0.9317 to 0.5521
T stage	− 0.7547	0.2572	0.0033	− 1.282 to − 0.2665
NLR	0.01952	0.02964	0.5102	− 0.03322 to 0.09822
PLR	0.0005021	0.001541	0.7446	− 0.002589 to 0.003589
MLR	− 0.3837	0.7915	0.6278	− 2.010 to 1.111
Hemoglobin	0.006610	0.01246	0.5957	− 0.01776 to 0.03162

*NLR* neutrophils/lymphocytes, *PLR* platelets/lymphocytes, *LMR* lymphocytes/monocytes

### Evaluation of efficacy prediction model of EGFR-positive NPC IC sequential CCRT combined with targeted therapy

**Fig. 2:**
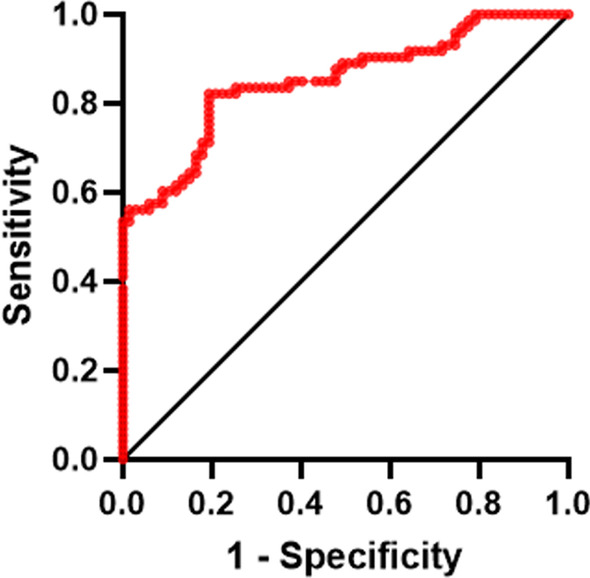
ROC curve of EGFR-positive NPC IC sequential CCRT combined with targeted therapy

**Fig. 3:**
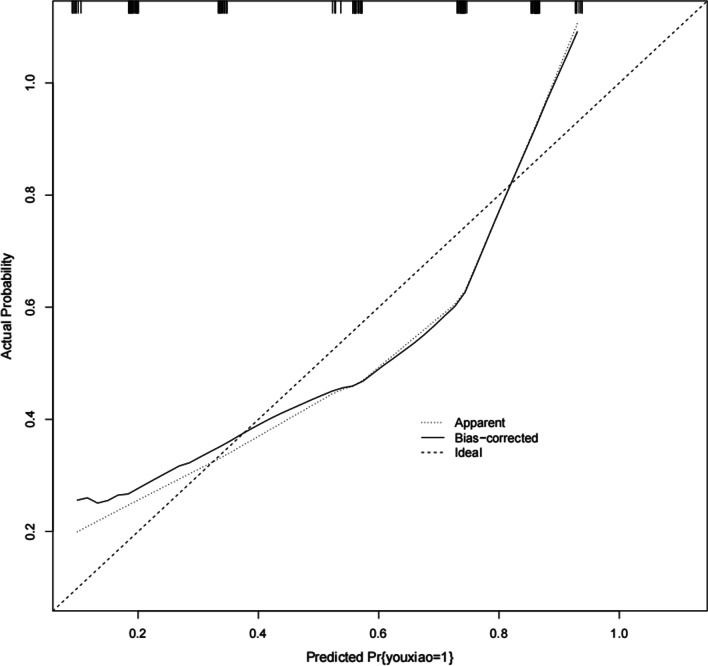
Calibration curve

**Fig. 4:**
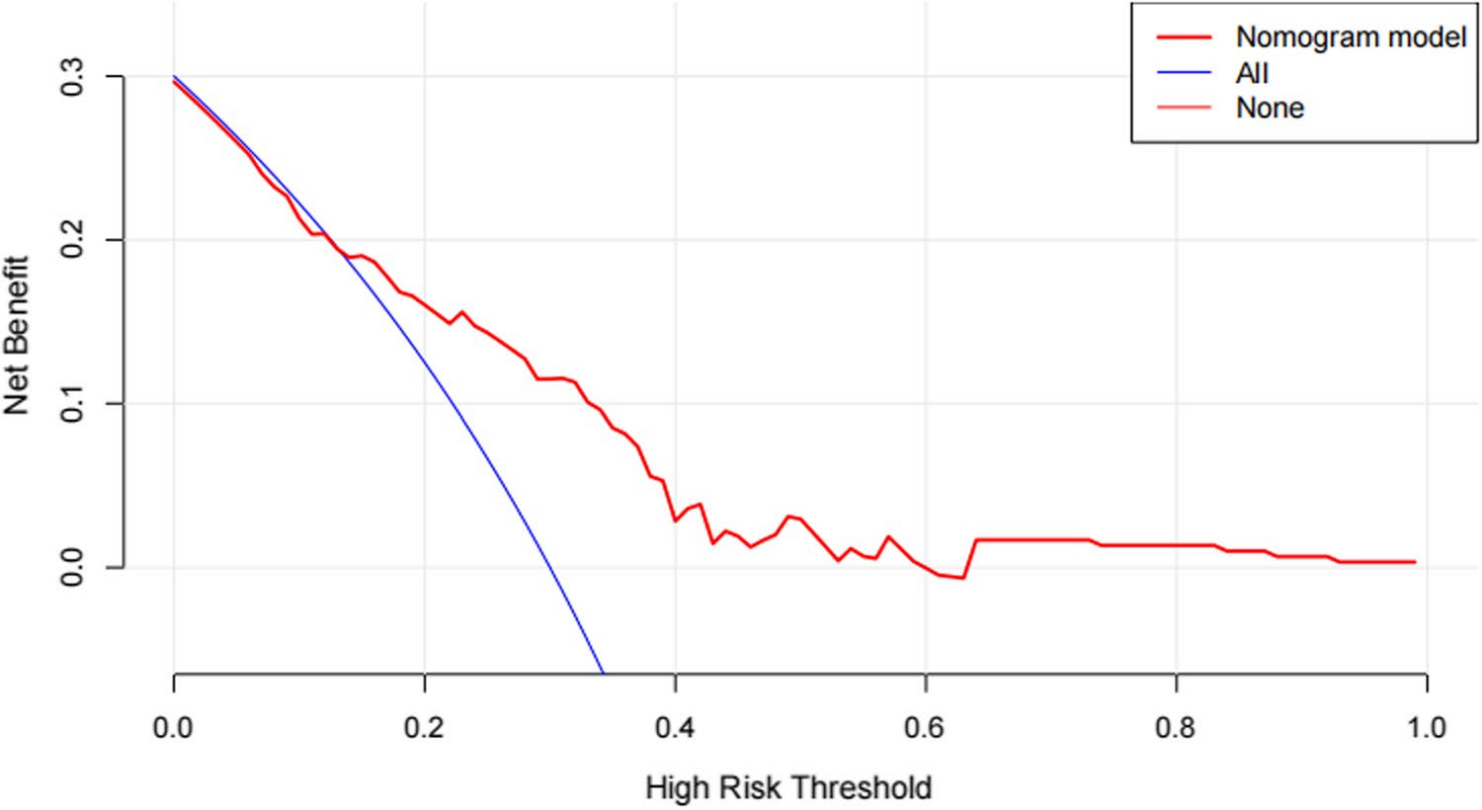
DCA curve

The Bootstrap method was used to resample 1000 times and perform internal verification of the nomogram, and the C-index was 0.8384. When cut-off = 0.5, the AUC is 0.8384 (*p* < 0.001) (Fig. 2), the 95% CI is 0.770–0.9068, the Hosmer–Lemeshow fit test X2 = 12.39, *p* = 0.1345(*p* > 0.05), and the prediction model accuracy is 72.58% (Table 4).The calibration curve shows a good fit between the predicted and true values (Fig. 3), confirming the good accuracy of the predictive model. The DCA curve indicates that the model has significant clinical applicability (Fig. 4).

**Table 4:** Total prediction accuracy of predictive performance model (cut-off = 0.5)

Classification table	Forecast number of non-CR	Forecast number of CR	Total number	Forecast accuracy (%)
Cut-off = 0.5				
Observe the number of non-CR people	30	22	52	57.69
Number of people observing CR	12	60	72	83.33
Total	42	82	124	72.58

## Discussion

In Southeast Asia and southern China, nasopharyngeal carcinoma (NPC) is one of the common tumors in the head and neck [15]. Its annual incidence is roughly 50/100,000, and the majority of patients were diagnosed at an advanced stage [16]. Despite recent advances in diagnosis and treatment, the prognosis for NPC patients remains very poor. New agents are urgently needed to improve outcomes. Targeted therapy is a hot spot in tumor treatment in recent years and the epidermal growth factor receptor (EGFR) is a promising new therapeutic target for the treatment of cancer. EGFR is expressed in 85% of NPC and correlates with a more aggressive phenotype, greater resistance, and poor prognosis. Therefore, for EGFR-positive advanced NPC, anti-EGFR-targeted therapy is considered a potential adjunct to IC sequential CCRT [17, 18].

In this study, we successfully developed a new model using a nomogram for predicting the efficacy of EGFR-positive NPC who have received IC, and CCRT combined with targeted therapy. The model shows that for patients with EGFR-positive NPC, the use of targeted therapy can improve the prognosis of efficacy. For patients with T1 and T2 stages, IC combined with CCRT has a good effect, and the addition of targeted drugs can significantly improve the treatment response rate on this basis. For patients with T3 and T4 stages, the efficacy of IC combined with CCRT is not good, and the addition of targeted drugs can significantly improve their CR response rate.

A number of previous studies have investigated the prognosis of NPC, Li et al. [19] constructed a prognostic nomogram model for NPC, but based only on blood biomarkers and clinical characteristics, without innovative inclusion of other prognostic factors. Wnag et al. [20] only examined the efficacy of targeted drugs for advanced NPC alone and did not combine multiple prognostic factors to construct predictive models to predict their efficacy. We innovatively incorporated targeted drug therapy and used a nomogram model to predict the response rate to targeted therapy in patients with EGFR-positive NPC compared to other prognostic models for NPC. Early studies have demonstrated the clinical efficacy of targeted therapies in the treatment of NPC, but many of these studies are still at the preclinical or early stage of research. Tumor treatment is becoming more precise and personalized, and targeted therapy has the potential to become an adjuvant therapy. By building a predictive model, a comprehensive review with predictors can be used to more accurately select the beneficiary population. As shown by the internal composition of this study nomogram, for EGFR-positive NPC patients, the use of targeted drugs can further increase the CR rate in patients with T1 and T2 stages with better efficacy of IC sequential CCRT. It can also improve the CR efficiency in patients with T3 and T4 stages who have poor efficacy of IC sequential CCRT. It is recommended that patients with EGFR-positive advanced NPC be treated with targeted combination therapy as early as possible, regardless of T stage.

In this study, based on the clinical data of EGFR-positive NPC patients, the equation was constructed: *Y* = 0.3199 + 2.494X1 + − 0.7547 × 2 (*Y* = efficacy, X1 = targeted drug therapy, X2 = T stage). A nomogram prognosis prediction model was established. Model results provide preliminary assessment of efficacy of IC sequential CCRT and targeted therapy in patients with advanced EGFR-positive NPC. The model’s internal composition allows scoring EGFR-positive NPC patients for targeted therapy efficacy. For high-response populations, targeted therapy is highly recommended, which improves the precision and personalization of tumor treatment. This significantly improves clinician decision-making and patient prognosis.

Since this is a retrospective study, there is a possibility of bias in the selection of cases. In addition, this study was a single-center study with a limited number of patients and lacked a broadly meaningful external validation cohort. A multicenter study with a larger sample size and external validation should be conducted.

## Conclusion

In conclusion, the study innovatively constructed a prediction model for IC sequential CCRT in combination with targeted therapy in EGFR-positive NPC patients. The model is able to predict the efficiency of patients on targeted therapies before treatment. Targeted therapy is recommended for patients with EGFR-positive NPC at T3 and T4 stages with poor efficacy of IC sequential CCRT to improve their prognosis and survival rate.

## Data Availability

Data of this study are available from the corresponding author on reasonable request.
